# Welcome to *Viruses*: A New Open-Access, Multidisciplinary Forum for Virology

**DOI:** 10.3390/v1010001

**Published:** 2009-04-01

**Authors:** Eric O. Freed

**Affiliations:** Virus-Cell Interaction Section, HIV Drug Resistance Program, National Cancer Institute at Frederick Frederick, MD, USA; E-mail: efreed@mail.nih.gov; Tel. 301-846-6223; Fax: 301-846-6777

The field of virology has never been more exciting as a research area and more relevant to human health as it is in 2009. The AIDS pandemic, caused by the uncurtailed spread of HIV-1 in large parts of the world, continues to have an enormous impact on the human condition. The threat of a global outbreak of highly pathogenic avian influenza remains real, and memories of the devastation created by the SARS coronavirus are still fresh. While many of the world’s most lethal viruses (Ebola, Hendra, Rift Valley fever, etc.) are geographically contained, the possibility of deliberate transmission of such infectious agents as biological weapons is cause for concern. Increased understanding of all viruses, not only these “newsworthy” pathogens, is warranted as it is impossible to predict the origins of the next viral epidemic. Increased human movement, global climate change, and disruption of natural ecosystems all favor the transmission and spread of both established and emerging viruses. Agricultural interests world-wide continue to be significantly impacted by viral agents.

In addition to the obvious significance to human health and welfare, the study of viruses continues to expand our knowledge across a broad range of biological disciplines from immunology, cell biology, and molecular biology to evolutionary biology. Viruses are also in high demand as tools for gene delivery, in the setting of both the laboratory and the clinic. In this era of nanotechnology, viruses offer important insights into elegant design and assembly principles.

Although I think no one can dispute the importance of virology, as the first Editor-in-Chief of *Viruses* I am faced with the question of whether we really need another journal devoted to this topic. While I feel that the answer is yes, it will doubtless be challenging to carve out a niche for this new journal given the number of well-established virology journals already in existence. The strength of a journal is inevitably determined by the quality of manuscripts that it receives and the rigor of its review process. *Viruses* has assembled a stellar editorial board that will encourage contributions from leading investigators and ensure the quality of all manuscripts that are published. At a time when publication in major journals can take months after a paper is accepted and can cost a significant portion of a small laboratory’s research budget, the open-access format of *Viruses* will allow rapid publication without undue delay or expense.

To get *Viruses* off the ground, we are planning an ambitious series of special issues devoted to virology topics of particular interest and importance. These include issues on influenza, pandemics and vaccinations; AIDS vaccines; antiviral responses to herpes viruses; hepatitis viruses; HIV drug resistance; interferon antiviral response and viral evasion; newly identified respiratory viruses; novel viral vector systems for gene therapy; pathogenesis of emerging and re-emerging RNA viruses; polydnaviruses and other insect viruses; retroviral enzymes; subviral RNAs; and virus dynamics and evolution. These issues will be edited by leaders in their respective fields, again helping to increase the profile of the journal and the quality of its publications.

I expect *Viruses* to become a venue for the rapid and affordable publication of high-quality virology manuscripts from scientists around the world. I, and all members of the editorial board, extend our welcome to this new journal and look forward to hearing from you and learning about your research.

